# Statistical Issues in the Analysis of ChIP-Seq and RNA-Seq Data

**DOI:** 10.3390/genes1020317

**Published:** 2010-09-27

**Authors:** Debashis Ghosh, Zhaohui S. Qin

**Affiliations:** 1 Department of Statistics and Public Health Sciences, Penn State University, 514A Wartik Building, University Park, PA 16802, USA; 2 Department of Biostatistics and Bioinformatics, Rollins School of Public Health, Center for Comprehensive Informatics, Emory University, 1518 Clifton Rd., N.E., Atlanta, GA 30322, USA; E-Mail: zhaohui.qin@emory.edu

**Keywords:** next generation sequencing, statistical analysis

## Abstract

The recent arrival of ultra-high throughput, next generation sequencing (NGS) technologies has revolutionized the genetics and genomics fields by allowing rapid and inexpensive sequencing of billions of bases. The rapid deployment of NGS in a variety of sequencing-based experiments has resulted in fast accumulation of massive amounts of sequencing data. To process this new type of data, a torrent of increasingly sophisticated algorithms and software tools are emerging to help the analysis stage of the NGS applications. In this article, we strive to comprehensively identify the critical challenges that arise from all stages of NGS data analysis and provide an objective overview of what has been achieved in existing works. At the same time, we highlight selected areas that need much further research to improve our current capabilities to delineate the most information possible from NGS data. The article focuses on applications dealing with ChIP-Seq and RNA-Seq.

## 1. Introduction 

**Figure 1:**
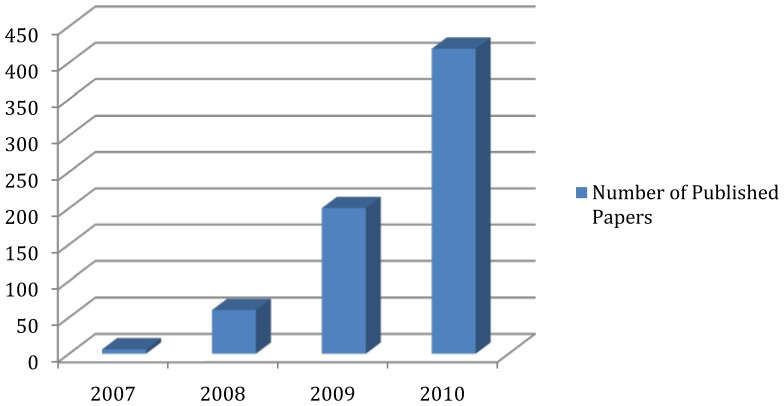
Number of publications by year deposited in PubMed on “Next generation sequencing” (Year 2010 figure is projected).

Much like the development of microarray technology for measuring gene expression in the late 1990s and early 2000s, the development of technologies for high-throughput sequencing, termed next-generation sequencing (NGS) technologies, is having an impact on the types of questions that biologists can ask these days. Already, these technologies have resulted in a multitude of high-impact studies with very diverse biological applications. These range from genome-wide survey of transcription factor binding sites: Chromatin Immunoprecipitation followed by high throughput sequencing (ChIP-Seq) [1,2,3,4], comprehensive surveying of the entire transcriptome: RNA-Seq [5,6] , global methylation patterns [7,8,9,10], sequencing the organisms present in a complex mixture [11], full genome sequencing of individuals and samples [12,13,14,15], and understanding the role between sequence variants and their effects on gene expression [16,17]. While the applications given so far have been applied to data from humans, NGS technologies have also been applied to data from model organisms, such as yeast [18], bacteria [19,20] the mouse [5] and ancient species [21,22]. This list of applications is brief and by no means exhaustive; it is also fair to say that the use of NGS technologies is on the steep part of the usage curve. Figure 1 shows the steady increase of number of publications related to the topic of “next generation sequencing”. One of the advantages of the technologies is that it allows single-investigator labs to generate data that was previously the domain of large-scale sequencing centers. 

Just as with the development of microarray data platforms a decade ago, this new technology raises important statistical problems, issues and challenges. Our goal in this article is to give a description of the NGS technology, outline an analysis pipeline that is typically used for data generated from NGS platforms and to highlight open areas and issues that need further exploration. Given the sheer volume of new articles that are coming out on NGS technologies, attempting to review the current state of the art is at best akin to aiming at a moving target. However, we hope that at the end of this article, statisticians, computer science researchers and data analysts have a better sense of the experiment that is performed to generate the data as well as issues involved in their analysis. 

## 2. Experimental Platform

We now give a brief overview of the NGS data generation platform. For more in-depth coverage of the technologies, the readers are referred to excellent reviews such as Mardis [23] and Metzker [24]. Although knowledge on technological detail is not required for statisticians focusing on high-level analytical issues, past experience has suggested that insights into the source of the data sometimes offer crucial advantages. 

There are three major steps involved in a typical DNA sequencing experiment. The first is that the DNA of the starting material is fragmented, followed by the ligation of adaptor sequences. Through some type of a polymerase chain reaction (PCR), these sequences become clusters of sequencing features that are to be processed by the sequencing machine. These features are then run through the sequencing machine, where a combination of biochemical and imaging steps are performed to generate the sequence reads.

The particular nature of the steps depends on the machine being used. The three major platforms that are currently in use are the Roche/454 platform which utilizes the pyrosequencing technology [25], the Illumina/Solexa Genome Analyzer which uses the sequencing by synthesis technology [26], and the Life Technology/Applied Biosystem sequencing by oligonucleotide ligation and detection (SOLiD) system. 

Sequencing platforms represent perhaps the fastest-evolving genomics technologies today. There are now multiple emerging “next-next-generation” sequencing technologies such as the true Single Molecule Sequencing (tSMS) technology from Helicos [27] and Single Molecule Real Time (SMRT) technology from PacBio [28]. They are often described as single molecule technologies. These new technologies offer even higher throughput with further reduced cost. Another key advantage is that these technologies no longer require PCR amplification in the sample preparation step. This eliminates a major source of bias. Although the underlying technologies are drastically different, their end result is very similar: a list of sequence reads. Although the read lengths from most NGS platforms tend to be shorter than those of the classical Sanger sequencing approach, this improved significantly in recent years. Despite this drawback, the sheer amount of high-quality sequences generated has proven to be invaluable in many applications, as alluded to in the Introduction. The raw data generated are imaging files corresponding to the intermediate sequencing steps, but they tend to be really large (on the order of terabytes per sequencing run) and therefore difficult to manipulate. Most labs discard them or ignore them in analysis. 

Another important advance in NGS is the development of the “paired-end” sequencing technologies. These are technologies that allow sequencing to occur on both ends of the same DNA molecule. The end result is a pair of two sequencing reads that are supposed to be only a short distance away from each other in the genome. This technology leads to a greater ability to resolve ambiguity of read mapping hence producing more and higher-quality data. The new technology is the most useful in detecting structure variants and performing *de novo* assembly. 

## 3. Mapping reads from NGS experiments

Once the reads are generated, a key issue is to map them to their correct genomic locations. Because reads generated from NGS technologies are much shorter than those generated from the classical Sanger sequencing, previously developed alignment algorithms will not work for NGS data. There are two situations of interest: (1) when a reference genome is available; (2) when no reference genome is available. In the case of (1), the algorithm problem is one of alignment, while for (2) the computational algorithm is one of assembly. Both areas have been intensely researched recently with+in the bioinformatics community. We focus on the situation (1); a recent review of algorithms for (2) can be found in Flicek and Birney [29]. 

While a computationally feasible and attractive algorithm for local sequence alignment is the Smith-Waterman algorithm [30], it has not been computationally feasible to use when analyzing millions of sequence reads. Thus, there has been many algorithms developed for mapping these reads to a reference genome: ELAND (Illumina Inc, San Diego, CA). Other alignment tools are MAQ [31], SOAP [32], ZOOM [33], BOWTIE [34], SeqMap [35], GSNAP [36], BFAST [37], PASS [38] and BWA [39]. There are also methods developed specifically for mapping the short color space reads that is unique in the SOLiD platform, such as SHRiMP [40]. As discussed nicely in a recent review by Flicek and Birney [29], most methods can be categorized into two types of approaches: (a) hash-table based approaches; (b) Burrows-Wheeler Transform (BWT)-based methods. The idea behind hash-table based approaches is to generate a data structure (hash table) that can index the sequence information in a way such that searching can be done rapidly. The hash table can be constructed using either the sequence reads from the experiment or the reference genome. Note that there is a tradeoff between the size of the hash table *versus* the speed of scanning the sequences against the hash table. 

The more recent development has been in the use of BWT methods. These techniques are based upon the concept of a suffix array created from sequence data that has been transformed using BWT, which allows for more efficient searching than a suffix array created from the original data. There are two steps involved. First, the reference genome is modified using the BWT. Then, the index is created. As has been seen for packages such as BWA [39], use of the BWT-based methods for alignments leads to increased gains in efficiencies of storage relative to the hash-table based approaches [41]. 

Typically, the alignments make use of the sequence reads. However, all sequencing technologies also provide mappability scores as part of the base calling outputs in its accompanying software. Ideally, the mappability score equals the phred-scaled probability of the read being wrongly mapped. More recent alignment algorithms have attempted to incorporate the quality scores into the alignment procedure [31]. We view this as an improvement in that incorporation of quality scores will allow for proper probabilistic assessment of read quality and mapping. 

## 4. Statistical methods for ChIP-Seq experiments

In this review, we focus on two types of experiments that can be done using the NGS technology. The first type of experiment is called ChIP-Seq (chromatin immunoprecipitation followed by direct sequencing). This experiment deals with understanding the global DNA binding pattern of regulatory proteins such as transcription factors (TFs). Understanding transcriptional regulation is one of the key challenges in molecular biology. One critical step during this process is to determine how proteins interact with target DNA to regulate gene expression. Biologists have been constantly searching for better techniques to detect *in vivo* protein-DNA interactions.

Prior to the availability of high-throughput sequencing, the technology of choice for identifying binding sites of TFs genome-wide was ChIP-chip [42,43], which couples the Chromatin Immunoprecipitation assay with array-based hybridization. The protein-DNA binding is recognized by detecting hybridization signals using a fixed set of probes on DNA microarrays. Using the ChIP-chip technique, scientists are able to uncover many new transcription factor (TF)-DNA interaction sites [42,43,44,45]. However, due to their restriction to the probes present on the DNA microarray, such methods are naturally limited in scale and resolution. Whole-genome tiling arrays are also expensive and technically challenging. 

Theoretically, the comprehensiveness and the high resolution are two key advantages of ChIP-seq over the ChIP-chip technique [4]. The above is mostly true for ChIP experiments on TFs: most of the ChIP-enriched regions identified from ChIP-chip assays are 1 kb or wider, whereas peaks identified from ChIP-Seq are typically less than 500 bp. Therefore, compared to control sequences of the same length, enrichment tests using the Chi-Square test often result in more significant results from ChIP-Seq. Multiple studies have demonstrated the improved motif enrichment under the peaks detected by ChIP-Seq compared to those found by ChIP-chip [2,4,46,47,48]. 

### 4.1. Detecting enriched regions. 

Given the mapped sequence reads, a common goal is to then identify regions/locations where there is signal present; we will term these as enriched regions or equivalently peaks. A completely *ad hoc* approach would be to take all locations where there is at least one sequence read found to be a peak. Such a heuristic does not account for the inherent variability that exists in the data. Towards that end, many statistical algorithms have been developed for the problem of peak finding in NGS data [2,4,46,49,50,51,52,53,54,55,56,57,58]. See also review and performance comparison studies papers on ChIP-Seq peak calling [59,60]. While there are many approaches available, we group them into three categories. The first class of methods involves taking a moving average of sequence reads within a fixed or variable-width window and scans the window through the entire genome. Then a randomization scheme or a nonparametric method is used to determine the null distribution of counts within the genome so that one can construct an estimate of the false discovery rate (FDR) [61]. The FDR is defined to be the number of expected false positives among a set of locations that are called as peaks. The FDR has been extensively used in microarray studies [62] and has become a standard metric for calibrating error rates in high-throughput genomic assays. A related concept to the FDR is the q-value [63], which estimates the minimum FDR at which a given peak would be called significant. For example, the following are the steps in the algorithm for the F-seq peak-calling algorithm [49]:

1. Compute a smooth estimate of the density of the tag counts using a nonparametric kernel density estimator based on a default window size that is guaranteed to be numerically stable;
2. Compute an average number of features for window *w* as *n_w _*= *nw*/*L*. Here, n is taken to be the number of sequence reads, w is the size of the window, and L is the length of the chromosome.
3. Calculate the kernel density at a fixed point, *x_c_*, within the window given a random and uniform distribution of the *n_w_* features.
4. Repeat step 2 *k* times to obtain a distribution of the kernel density estimates for *x_c_*. For large *k* the kdes become normally distributed.
5. The threshold is *s* SDs above the mean of this normal distribution.

A related algorithm to that proposed in F-seq is given in the QuEST algorithm [55]. 

The second class of methods use the same approach for finding peaks but then make inferences based on a probabilistic model in order to assess the significance of the peaks that have been found. Typically, what has been presumed has been a Poisson probability model, as the data that are generated from an NGS experiments are counts. The support of the Poisson distribution is the set of nonnegative integers, so this supports it as a natural model for the analysis of read data. For the Poisson distribution with mean parameter λ, the probability of observing at least R reads at a given location is given by

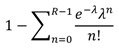


The assumption of the Poisson distribution has been questioned by many authors. Zhang *et al.* [56] found the assumption of one parameter λ to not match their empirical data. They proposed a local mean parameter that would be estimated for each peak separately. By definition, their approach will lead to stricter control of the FDR than using one value for the mean parameter. Another approach is to assume that the mean parameter is itself random. By definition, λ can be any nonnegative number. A natural distribution to use is the Gamma distribution. Combining the Gamma and Poisson distributions leads to a negative binomial distribution for the sequence counts, which is the null distribution that is used in the software packages CisGenome [64] and BayesPeak [51]. 

More complicated algorithms use Hidden Markov models (HMMs) to account for the spatial dependence in the peak counts at adjacent locations in the genome. A HMM is a probabilistic model that assumes latent states that are of scientific interest. HMM was originally developed by Baum and his colleagues in the 1960s. It was first applied to solve engineering problems such as speech recognition [65]. Given the latent states, another probabilistic model is assumed for the observed data (here the tag counts). The latent variables are also presumed to have a spatial dependence structure along the genome. HMMs have a long history in bioinformatics and genetics research [66,67,68,69,70,71,72,73,74,75], and their application to this setting seems quite appropriate as well. As is suggested, fitting HMMs tends to be more complicated than the previous approaches, although there is a computationally feasible optimization algorithm, the forward-backwards algorithm [76]. 

While this area continues to be an area of active research, we point out several issues that merit further attention by statisticians. The first is that not all reads are necessarily mappable; while Solexa technology provides mappability scores for the reads, they have mostly been used as a data quality filter in the analysis. Approaches that model the mappability score in terms of finding peaks would be an improvement, analogous to using them in the alignment problem as well. The second area of research is the calculation of FDR. Model-based approaches allow for natural estimation of FDR, but they are reliant on the modeling assumptions (e.g., Poisson/negative binomial experiments). Typically, permutation methods have been used to estimate a null distribution, but it is not clear how to permute in this situation. We expect there to be spatial correlations in the data so that care must be used in the resampling scheme. Resampling techniques from fields such as time series [77] might be of important use, but this has not been explored. The ideal setup would be to have additional samples available representing a control distribution; permutation of the sample labels can then be used to estimate the null distribution used to estimate FDR. However, more research is needed in the model-based procedure; one issue that has not been considered is that the data are fundamentally discrete, while most FDR-controlling procedures implicitly work with continuously distributed data. Finally, new methods of evaluating the performance of the various peak-calling procedures are needed. Given that the ultimate goal is some type of downstream analysis, it may be useful to compare methods based on the ultimate downstream goal. 

### 4.2. Follow–up analysis

Much research has been devoted to the aforementioned peak identification. However, plenty of work remains after this step. An important goal for the follow–up analysis is to offer clues and biological insights from the identified protein-DNA interactions. A summary of the binding pattern can be very informative for biologists. Such summaries may include distribution of distances from peaks to the transcription start site of the nearest gene; break down of genomic location annotation such as intron or untranscripted regions; enrichment of functional categories or pathways in all target genes that show evidence of binding. Several tools have already been developed for such purposes [78,79,80].

Another important question is whether there is any significantly over-represented sequence motif in the ChIP-enriched regions. It is well-known that many transcription factors (TFs) bind to the DNA in a sequence-specific manner [81,82], hence the recognition of enriched sequence motifs will offer significant implications on the transcription regulation of the TF. There are two ways to perform the motif analysis: *de novo* motif identification and known motif scan. Both are important problems in computational biology and have been intensively studied. A variety of different software programs have been developed over the past two decades [83,84,85,86,87,88,89]. See Tompa *et al.* [90] for a review on this topic.

One type of follow–up analysis from ChIP-Seq experiments involves performing a motif search for the regions that are declared to be peak regions. The motif corresponds to the DNA pattern that the TF binds to. Such an analysis is important in situations where the TF binding motif pattern is unknown. However, it is also useful when the canonical binding motif for the TF is known, as it will serve as a validation of the data and peak calling procedure. In general, we currently lack a comprehensive census of binding motif patterns for all transcription factors. There are various stored TF binding motif databases, a subset of which have been experimentally verified. Due to this lack of coverage, *de novo* motif searches on a large number of ChIP-Seq binding sites have the potential to refine the motif patterns of the TFBS. 

It seems natural to expect that exploiting the quantitative information provided by high-throughput genomic assays will lead to the development of motif-finding algorithms with better sensitivity and specificity. This has been seen in studies using microarray [91,92] and ChIP-chip [86,93] data. ChIP-Seq technology has demonstrated remarkable sensitivity and specificity in identifying protein-DNA binding loci across the entire genome with higher resolution and few constraints. Typically, thousands of DNA sequences are routinely being identified as putative candidates for harboring TF-binding motifs. 

In a recent study, Hu *et al.* devised a novel computational algorithm named Hybrid Motif Sampler (HMS) [47], specifically designed for TFBS motif discovery in ChIP-Seq data. The HMS algorithm combines stochastic sampling and deterministic “greedy” search steps into a novel hybrid iterative scheme to accelerate the computation. The authors also introduced a novel Bayesian model to account for unique features contained in ChIP-Seq data, using four different real datasets. 

Due to the ever-increasing popularity of the ChIP-Seq technologies, we anticipate that more advanced computer algorithms will be developed for better *de novo* motif finding using ChIP-Seq data. 

Another viable strategy is to assess the relative enrichment of all known TF binding motifs. A reasonable hypothesis is that compared to a set of random control sequences, functional motifs that are involved in the regulatory process of the TF tend to be over-represented above non-functional motifs in ChIP-enriched regions. To evaluate the enrichment one may scan the entire set of ChIP-enriched regions identified from ChIP-Seq experiments for occurrences of known TF binding motifs. The result is then compared to the motif scan results obtained from a set of size-matched random control sequences of the same length as comparison. Commonly used statistical tests such as Fisher’s exact test or Chi-square test can be applied to quantify the level of enrichment. Subsequently, either the test statistics or the associated p-values can be used to rank all tested motif patterns to see which motifs are ranked on top. Such a strategy has been used in real ChIP-Seq experiments and provides useful knowledge to uncover fresh biological insights [48]. 

### 4.3. Combining ChIP-Seq with ChIP-chip data

Although ChIP-Seq is a new and more powerful assay, ChIP-chip technologies have been widely used and is capable of exposing ChIP-enriched regions in parts of the genomes that most NGS technologies have difficulty to access (due to repeats or chromatin states). For a well-studied TF, it is often the case that ChIP-chip experiments on the same TF have been conducted and the data are publicly available. When this is the case, it is desirable to take advantage of the existing ChIP-chip data sets to supplement the ChIP-Seq data on hand to improve the comprehensive detection of protein-DNA interaction events. For example, Robertson *et al.* reported that the overlap between ChIP-enriched regions identified by ChIP-chip and ChIP-Seq is about 60% [2]. While such a joint analysis has promise, it is a challenging task to account for the heterogeneity of data from the ChIP-chip and ChIP-seq platforms. This is because the two technologies show vastly different behavior in terms of sensitivity and specificity. The peaks identified by ChIP-seq are much sharper and narrower than those in ChIP-chip due to its superior resolution. 

For inference using both sources of data, Choi *et al.* proposed a hierarchical hidden Markov model (HHMM) for an integrated analysis using both ChIP-chip and ChIP-Seq data [94]. To be specific, inference results from individual HMMs in ChIP-chip and ChIP-Seq experiments are summarized in a higher-level HMM. Analysis results from two well-studied TFs, NRSF and CTCF, suggested that HHMM produced improved TFBS identification comparing to analyses using individual data sources. In addition to statistical inference, it is also of interest to study the discrepancies in detecting ChIP-enriched regions using these two technologies, which may shed light on the limitation of these technologies and on their false positives and false negative rates. 

## 5. RNA-Seq experiments: measuring gene expression 

While microarrays have been widely used for the analysis of gene expression so far, it is possible now to apply NGS technologies to accomplish this task [95]. NGS promises the potential of giving actual counts for genetic elements as opposed to fluorescence intensities (either single- or dual-channel) that were generated using previous microarray platforms. This technology is called RNA-seq and has been utilized in several recent studies [5,6,16,17]. As with other NGS data, we expect that utilization of this technology will continue to expand in the future. 

While the NGS platforms are very different technically from gene expression microarrays, it is also true that we can learn much from the microarray literature. First, it was discovered that there existed various systematic biases in the intensity measurements that required various preprocessing and normalization methods. Similarly, we can expect that there will be biases that will exist with this type of data as well. Past studies have shown considerable bias in terms of sequenceability along the genome. There are many factors contributing to this bias such as GC content and repeat regions [96]. This again underscores the necessity of replication; while most experiments tend to have no replications, we expect that replication will begin once technology costs decrease. 

Another new consideration is that this type of data will lead to more consideration of statistical methods for discrete data. This again is in strong contrast with gene expression data from microarray platforms, where the measurements were typically modeled as having continuous distributions. This will have impact on the types of analyses that are being done. For example, one analysis that is done frequently using high-dimensional gene expression microarray data is to find genes that are differentially expressed between two or more experimental conditions or treatment groups (e.g., differentially expressed in cancerous tissue relative to healthy tissue). This leads into issues of multiple comparisons and simultaneous inference because the number of genes being tested is on the order of thousands so that performing this many tests of hypotheses leads to the usual multiple testing problem. As discussed earlier, the standard adjustment in microarray analysis has been based on the false discovery rate, either via the q-value [63] or the Benjamini-Hochberg procedure [61]. This theory assumes that the test statistics have a continuous distribution; this will no longer be the case when the data consists of read counts, especially for tags that have small counts. There has been some literature studying multiple testing procedures for discrete data [97,98,99] and Ghosh 2010 [100], but these methods are very much in their infancy. 

Another procedure that has become quite common is to use gene set enrichment analysis methods [101,102] for the analysis of lists of selected/interesting genes that are obtained from microarray experiments. This typically involves comparing the intersection of the list of genes with an *a priori* defined group of genes (e.g., genes involved in cell-cycle metabolism) with the expected amount of overlap based on chance. There are many software packages available to do this calculation, a recent summary of which can be found in Ackermann and Strimmer [103]. However, doing this same analysis with NGS data poses certain issues. This is investigated nicely by Oshlack and Wakefield [104] and Young *et al.* [105] in which they find a length bias, *i.e.* longer tags are preferred in differential expression analyses of RNA-seq data. They propose standardizing by tag length to adjust for this bias. 

Another important application of RNA-Seq is to detect alternative splicing and splice junctions. Splicing is a post-transcriptional modification in which regions of RNA called exons are joined. At the same time, other regions of RNA called introns are removed. This leads to the mature messenger RNA that is subsequently translated into a protein. Understanding the nature of splicing events is difficult to achieve using microarrays. Deep coverage and the ability to interrogate the entire transcriptome with little restriction using sequencing has enabled the discovery of many splicing events and associated products, termed isoforms. A suite of software tools have been developed such as TopHat, Cufflinks and Scripture [106,107,108]. We expect this to continue to be an active research area. However, at the current time, microarrays will still be used as a less expensive and more rapid technology for gene expression measurements.

## 6. Experimental design considerations

While much attention has been given to the analysis of data arising from next-generation sequencing experiments, much less attention has been focused on the design of such experiments. In fact, there are two issues when it comes to samples. The first is the number of samples to use. Most experiments tend to be single-sample experiments (e.g., one transcription factor) [2,4], although some studies have available multiple samples [48,109]. Statistically, replication is always a desirable thing, as it allows for increased power for finding peaks.

A more intriguing issue is determining how many sequencing tags to obtain. This is related to how much sequencing depth to obtain for a sample. If the size of the genome is known, then one typical recourse is to the theory of Lander and Waterman [110] to determine the amount of sequencing coverage needed. For instance, at 1X coverage (total length of fragments = genome size), about 63% of the genome is covered. Besides covering more of the genome, increased sequence depth permits correction of sequencing errors. However in a metagenomic setting, or in situations where the reference genome is not known, then it is much more difficult to determine how much sequencing to do. In addition, one has to deal with the issue of only a fraction of reads being mappable. Finally, as noted earlier, we expect there to be biases due to factors such as the mappability of the reads, GC content and other factors. Because such biases are shifting constantly in next-generation sequencing, it is important to determine biases regularly. The major work in this area has been that of Wendl and Wilson [111]. However, this is clearly an area where statisticians have much to contribute. 

## 7. Conclusion and Future Directions

Similar to the microarray technologies 10 years ago, the ultra-high-throughput DNA sequencing technologies [25,26,112] are making the transition from development to widespread application rapidly. These new technologies make possible unbiased genome-wide analyses at single-base resolution. Therefore, genes and regulatory pathways involved in key biological process can now be more effectively examined. NGS technologies have enormous potential and will likely play a central role in furthering our understanding of fundamental biology and human diseases. Analogous to DNA microarrays, research efforts are likely to shift from technologies to extracting biologically and clinically insights using them. A wide variety of applications of NGS has or is going to emerge. With ever-improving technology and steady decline of sequencing cost, the amount of data generated using this technique is likely to rise sharply. The massive amount if data produced poses analysis challenges. There is wide-spread speculation that the cost of sequencing will be lower than the cost of storing and analyzing the sequenced data. To match the advances provided by the NGS technologies, significant attention and efforts have to be directed to the statistics and bioinformatics front. Sophisticated and tailor-made data analysis approaches will likely play a key role in fully realizing the power of the next generation sequencing technologies.
